# Intracoronary physiological indices for coronary microvascular dysfunction: from concept to clinical application

**DOI:** 10.1007/s12928-026-01252-8

**Published:** 2026-06-02

**Authors:** Tadashi Murai, Tsunekazu Kakuta, Kiyoshi Hibi, Tim P. van de Hoef

**Affiliations:** 1 Cardiovascular Center, Yokosuka Kyosai Hospital, Yokosuka, Japan; 2 Department of Cardiovascular Medicine, Tsuchiura Kyodo General Hospital, Tsuchiura, Ibaraki Japan; 3 Department of Cardiology, Yokohama City University Graduate School of Medicine, Yokohama, Japan; 4 Department of Cardiology, University Medical Centre Utrecht, Heidelberglaan 100, Utrecht, 3584 CX The Netherlands

**Keywords:** Coronary microvascular resistance, Coronary flow reserve, Coronary microvascular dysfunction

## Abstract

The coronary microcirculation has long been considered a “black box” due to the difficulty of its direct assessment. Abnormalities in this compartment, termed coronary microvascular dysfunction (CMD), are now recognized as important contributors to myocardial ischemia and are increasingly implicated across a wide range of cardiovascular diseases, underscoring the need for precise diagnosis and targeted treatment. Over the past two decades, invasive physiological assessments (IPA) performed during coronary catheterization have been developed to evaluate coronary microcirculation and provide a unique opportunity to assess patient-specific pathophysiology of CMD. IPA is now expected to play a pivotal role not only in the diagnosis of CMD, but also in risk stratification and the development of tailored therapeutic strategies. This article provides an overview of currently available intracoronary physiological indices, summarizing their clinical applications, strengths, and limitations.

## Introduction

Myocardial ischemia, caused by an imbalance between myocardial oxygen supply and demand, not only impairs quality of life but also increases the risk of adverse cardiovascular events [1]. Epicardial coronary artery disease (CAD) has long been considered the primary cause of myocardial ischemia, and the advent of coronary angiography (CAG) in 1958 marked a major milestone in the diagnosis of CAD [2]. As a result, conventional assessments and treatments for myocardial ischemia have mainly focused on CAD. However, shortly after the introduction of CAG, in 1967, Likoff reported a paradoxical subset of patients with chest discomfort and abnormal electrocardiograms but normal coronary angiograms [3]. This observation raised the possibility of an underlying coronary microvascular disorder—inherently elusive and difficult to visualize—as a cause of myocardial ischemia. Over the years, this condition gained recognition as “Syndrome X” in 1973 and later as “microvascular angina (MVA)” in 1988 [4, 5]. Today, the term “ischemia with no obstructive coronary arteries (INOCA)” serves as a broad concept encompassing myocardial ischemia in the absence of CAD, which includes both coronary microvascular dysfunction (CMD) and coronary spasm as underlying etiologies for myocardial ischemia [6]. 

Despite more than five decades of recognition that microvascular abnormalities or coronary spasm may contribute to myocardial ischemia, comprehensive evaluation of these mechanisms has remained limited in routine clinical practice worldwide. In Japan, coronary spasm provocation testing has been widely adopted in clinical practice earlier than in many Western countries, likely driven by both the higher prevalence of coronary spasm in Asian populations and the early publication of the world’s first guideline dedicated to coronary spasm in 2008, however CMD evaluation largely lagged behind [7]. Meanwhile, substantial progress has been made in invasive physiological assessment (IPA), which enables CMD evaluation by using intracoronary pressure and flow measurements, as well as their reactivity to vasoactive stimuli, obtained during coronary catheterization [8–11]. A paradigm shift occurred in 2018 with the publication of the CorMicA trial, the first study to demonstrate the clinical benefit of a pathophysiology-guided management strategy in suspected INOCA [12]. This landmark trial emphasized the clinical utility of IPA for stratification and laid the foundation for its integration into routine practice, alongside coronary spasm provocation testing.

The use of IPA and coronary spasm provocation testing, in combination referred to as coronary function testing or functional coronary angiography, offers unique advantages by enabling a comprehensive evaluation of vasomotor function of both epicardial and microvascular compartments in a single procedure. IPA plays a central role in this approach, providing quantitative physiological indices that allow functional evaluation of CAD and enable detection of CMD. Notably, IPA can be readily implemented in catheterization-capable hospitals as part of routine clinical workflows. This review highlights current invasive techniques for assessing CMD, outlining the development of key physiological indices, their clinical relevance, limitations, and potential applications.

## Pathophysiology of CMD

**Fig. 1:**
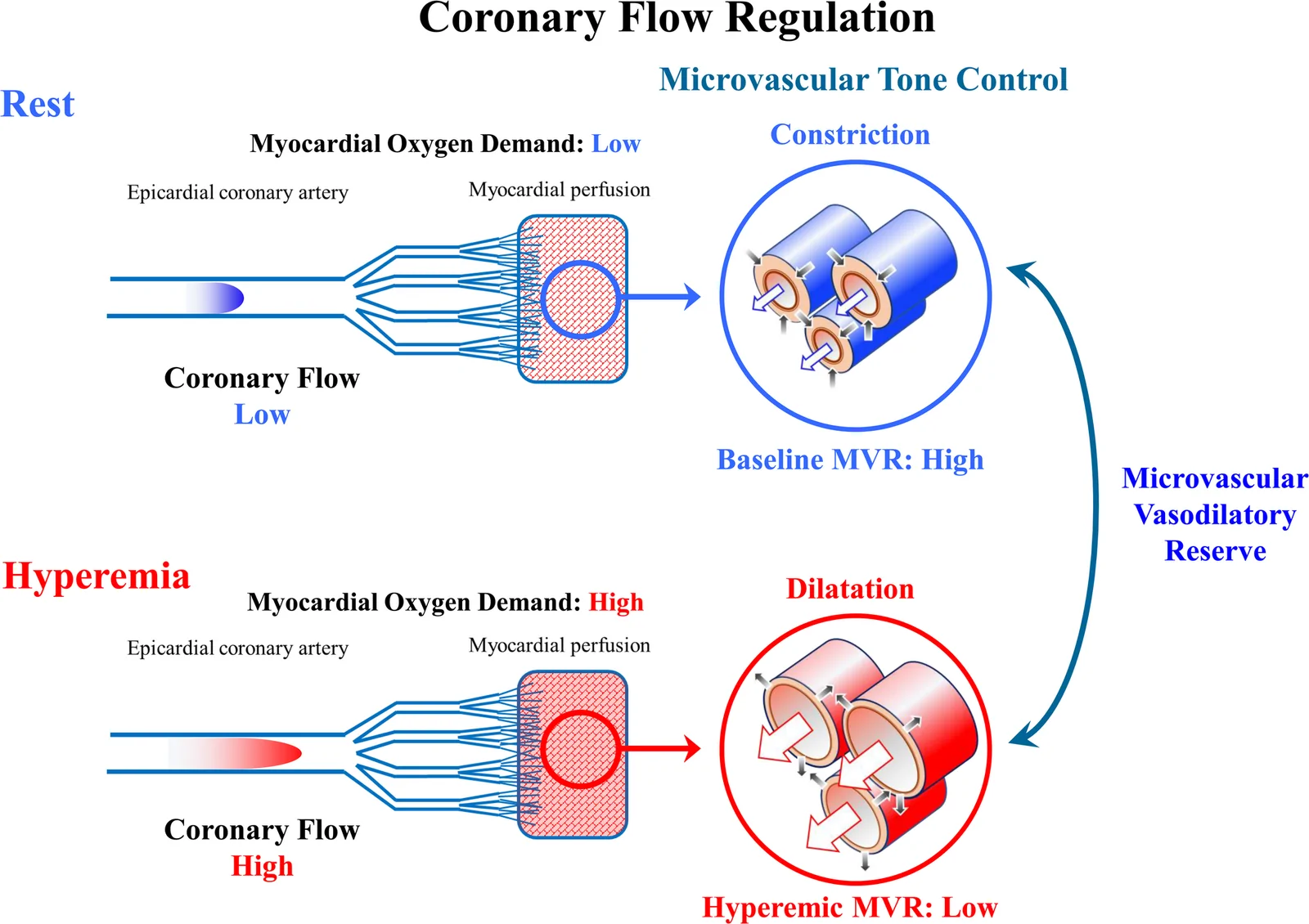
Coronary Microvascular Tone and Coronary Flow Regulation. This figure illustrates how coronary microvascular tone dynamically regulates coronary blood flow in response to myocardial oxygen demand. At rest, myocardial oxygen demand is low, and the microvasculature remains relatively constricted, leading to high baseline microvascular resistance (MVR) and low coronary flow. In contrast, during hyperemia, increased oxygen demand induces vasodilation, resulting in reduced hyperemic MVR and augmented coronary flow. This dynamic modulation of microvascular tone represents the physiological basis of microvascular vasodilatory reserve

The coronary arterial circulation is typically divided into two compartments with distinct vessel sizes and functions. The distal compartment, referred to as the coronary microcirculation, comprises resistive vessels smaller than 500 μm in diameter, including pre-arterioles (100–500 μm), intramural arterioles (< 100 μm) and capillaries. This compartment is responsible for size-dependent, layered regulation of myocardial blood flow in response to coronary flow, pressure, and metabolic demand, and accounts for more than 70% of total coronary vascular resistance [9, 13]. Since myocardial metabolism is predominantly aerobic and characterized by high baseline oxygen extraction, there is an almost linear relationship between oxygen demand and coronary blood flow [14]. In healthy individuals, increased myocardial oxygen demand is met by up to a five-fold increase in coronary blood flow, primarily achieved through a reduction in microvascular resistance (MVR) (Fig. 1) [9]. An imbalance between myocardial oxygen supply and demand may result from impaired regulation of vasomotor tone and/or a reduced capacity to augment coronary microvascular blood flow. These impairments are driven by various mechanisms, including both functional and structural abnormalities of the coronary microcirculation [10, 15]. 

1. **Functional disorder of the coronary microcirculation**.

- Impaired microvascular dilation and/or microvascular spasm due to endothelial dysfunction or hyperreactivity of vascular smooth muscle cells.
- Proposed mechanisms include increased oxidative stress, chronic inflammation, autonomic dysregulation, and other factors.

2. **Structural abnormalities of the coronary microcirculation**.

- Adverse alterations in the microvasculature, including:
  - Inward remodeling of the vessel wall.
  - Arteriolar and capillary rarefaction (i.e., reduced microvessel density).
  - Perivascular fibrosis or infiltration.
  - Increased extramural compression.
  - Microvascular plugging, etc.
- These structural abnormalities are thought to contribute to the pathogenesis of myocardial ischemia not only in MVA, but also in cardiomyopathies, systemic diseases (e.g., amyloidosis), and heart failure, particularly heart failure with preserved ejection fraction (HFpEF).

Furthermore, these abnormalities may increase the heterogeneity of local myocardial supply–demand mismatch, resulting in a patchy distribution of perfusion abnormalities. This characteristic of CMD may hinder its detection by conventional imaging modalities, because their spatial resolution is often insufficient to capture such small, localized perfusion defects [8, 14, 16]. Given the heterogeneity and multifactorial nature of CMD, both its pathophysiological understanding and accurate clinical characterization remain major challenges. In this context, IPA provides a valuable means to functionally assess CMD and stratify patient risk, offering a promising approach to address this unresolved clinical issue.

## Invasive physiological techniques for the assessment of coronary microcirculation

### Wire-based assessments of coronary blood flow

Various physiological indices have been proposed for evaluating the coronary microcirculation, and the cornerstone of invasive assessment is the measurement of coronary flow. Classic angiography-based methods, such as the TIMI flow grade, TIMI frame count, and TIMI myocardial perfusion grade, provide simple and semi-quantitative approaches [17]. However, these methods are inherently less objective and have notable limitations in precision. Although TIMI frame count is the most quantitative among angiography-based flow surrogates, several studies have shown that it demonstrates consistently poor correlation with coronary flow and microvascular function, whether assessed by invasive or non-invasive modalities [18, 19]. Accordingly, TIMI frame count should be regarded only as a supportive angiographic flow surrogate. Therefore, for more robust and quantitative assessments of coronary flow, three major wire-based techniques have been developed, offering advanced tools to evaluate coronary microcirculation. In addition, new technology is currently in advanced stages of development.

#### Intracoronary doppler flow assessment

The Doppler flow velocity sensor-equipped wire, introduced in 1992, enables the direct measurement of intracoronary flow velocity [20]. Later iterations of the technology – a 0.014-inch dual sensor-tipped intracoronary guidewire (ComboWire, Philips Volcano, Rancho Cordova, CA) – allowed simultaneous measurement of both average peak flow velocity (APV) and distal coronary pressure (Pd), enabling the calculation of physiological indices such as coronary flow reserve (CFR) and hyperemic microvascular resistance (HMR) [21]. This technique also facilitates detailed phasic analysis of flow dynamics, providing separate evaluations of systolic and diastolic phases, as well as forward and backward flow components. Such comprehensive analysis enables advanced assessments of microvascular function, including diastolic-limited indices and wave intensity analysis [22]. However, obtaining an adequate flow signal requires precise alignment of the Doppler sensor with coronary flow, making the procedure relatively technically challenging, especially in tortuous or anatomically complex vessels. These technical challenges have limited its widespread adoption in clinical practice. As of now, there is no armamentarium available for Doppler flow velocity measurements in clinical practice. New iterations of the technology are eagerly awaited, potentially overcoming prior technical limitations and advancing the potential of Doppler flow velocity assessment in routine clinical practice.

#### Thermodilution-based flow estimation

The thermodilution-based flow estimation method, introduced in 2001, uses a guidewire equipped with both pressure and temperature sensors (PressureWire, Abbott, St. Paul, MN) [23]. This is currently the most widely adopted invasive modality for coronary flow assessment in contemporary clinical practice and is positioned as a central method in recent clinical guidelines, including the 2023 JCS/JSCVS/JCC Guideline on Coronary Physiology [24]. When saline (2 or 3 mL) at room temperature is manually injected into the coronary artery from the guiding catheter, the guidewire shaft detects changes in electrical resistance, acting as a proximal sensor to register the start of the injection. In the distal coronary artery, a distal thermistor measures the temperature change caused by the arriving saline bolus. The guidewire should be advanced at least 6 cm into the coronary artery to allow adequate mixing of blood and saline before reaching the distal sensor. The interval required for the saline bolus to travel from the tip of the guiding catheter to the distal thermistor is measured as the mean transit time (Tmn), which is inversely proportional to coronary flow. Conventionally, three consecutive intracoronary saline injections are performed, and the average of these measurements is used to ensure reliability. This method is widely accessible; however, Tmn represents a surrogate marker of coronary blood flow rather than a direct measurement, with several inherent limitations [25]. Although the thermodilution technique provides seemingly robust measurements, its accuracy can be affected by multiple procedural factors. Accordingly, the correlations between this thermodilution technique and Doppler flow velocity or positron emission tomography (PET)-derived metrics of coronary blood flow are moderate. To ensure reliable measurement, it is critical to maintain wire stability, avoid excessive force during saline injection, and prevent guiding catheter-induced coronary occlusion. To address these limitations and enhance measurement reproducibility, the CATHCMD algorithm, published in 2024, provides a standardized workflow for thermodilution-based assessment and has facilitated broader clinical implementation of CMD evaluation [26]. Despite its inferior performance compared with Doppler velocity-based measurements, the broad clinical availability of thermodilution-based assessment supports its use as a practical tool for estimating coronary flow [23, 27]. 

#### Continuous thermodilution technique for absolute coronary flow measurement

A novel thermodilution-based volumetric measurement of coronary blood flow has been introduced [28]. This method utilizes the thermodilution technique with continuous saline infusion via a dedicated infusion microcatheter (RayFlow, Hexacath, France) that is advanced over a temperature-sensitive pressure monitoring guidewire (PressureWire X). Absolute blood flow (Q) is calculated using the known saline infusion rate (Qi), saline infusion temperature (Ti), baseline blood temperature (Tb), and the temperature of the blood mixed with saline in the downstream coronary artery (T), as follows:

$$\mathbf{Q}\hspace{0.17em}=\hspace{0.17em}\mathbf{Q}\mathbf{i}\times\left[\right(\mathbf{T}\mathbf{b}-\mathbf{T}\mathbf{i})/(\mathbf{T}\mathbf{b}-\mathbf{T}\left)\right]\times1.08$$

When Tb is set to 0, and Ti and T are expressed as deviations from Tb, the equation simplifies to:

$$\mathbf{Q}\hspace{0.17em}=\hspace{0.17em}\mathbf{Q}\mathbf{i}\times(\mathbf{T}\mathbf{i}/\mathbf{T})\times1.08$$

This provides an accurate, operator-independent measurement of absolute blood flow in mL/min and has been validated in human subjects [28, 29]. A recent study has reported a physiologically interesting reaction of the coronary circulation to continuous saline infusion using the dedicated infusion microcatheter, where a room-temperature saline infusion at 20 mL/min was noted to induce stable maximal hyperemia, whereas a 10 mL/min infusion maintained resting flow conditions [30, 31]. It is hypothesized that the infusion of saline through the 4 sideholes at the end of the dedicated infusion microcatheter induces intravascular hemolysis, leading to a local release of vasodilatory compounds, like ATP, which is ultimately responsible for localized microvascular vasodilation [32]. This feature enables CFR assessment without the use of potent vasodilators like adenosine. Moreover, simultaneous Pd measurement allows for the calculation of absolute MVR. Given its potential to standardize coronary flow measurements, this method is expected to become increasingly incorporated into clinical practice. Its main limitation currently remains the necessity for additional armamentarium such as a suitable infusion pump, and the costs of the dedicated infusion microcatheter that can be considered prohibitory for widespread application.

### Physiological Indices for evaluating coronary microvascular function

**Fig. 2:**
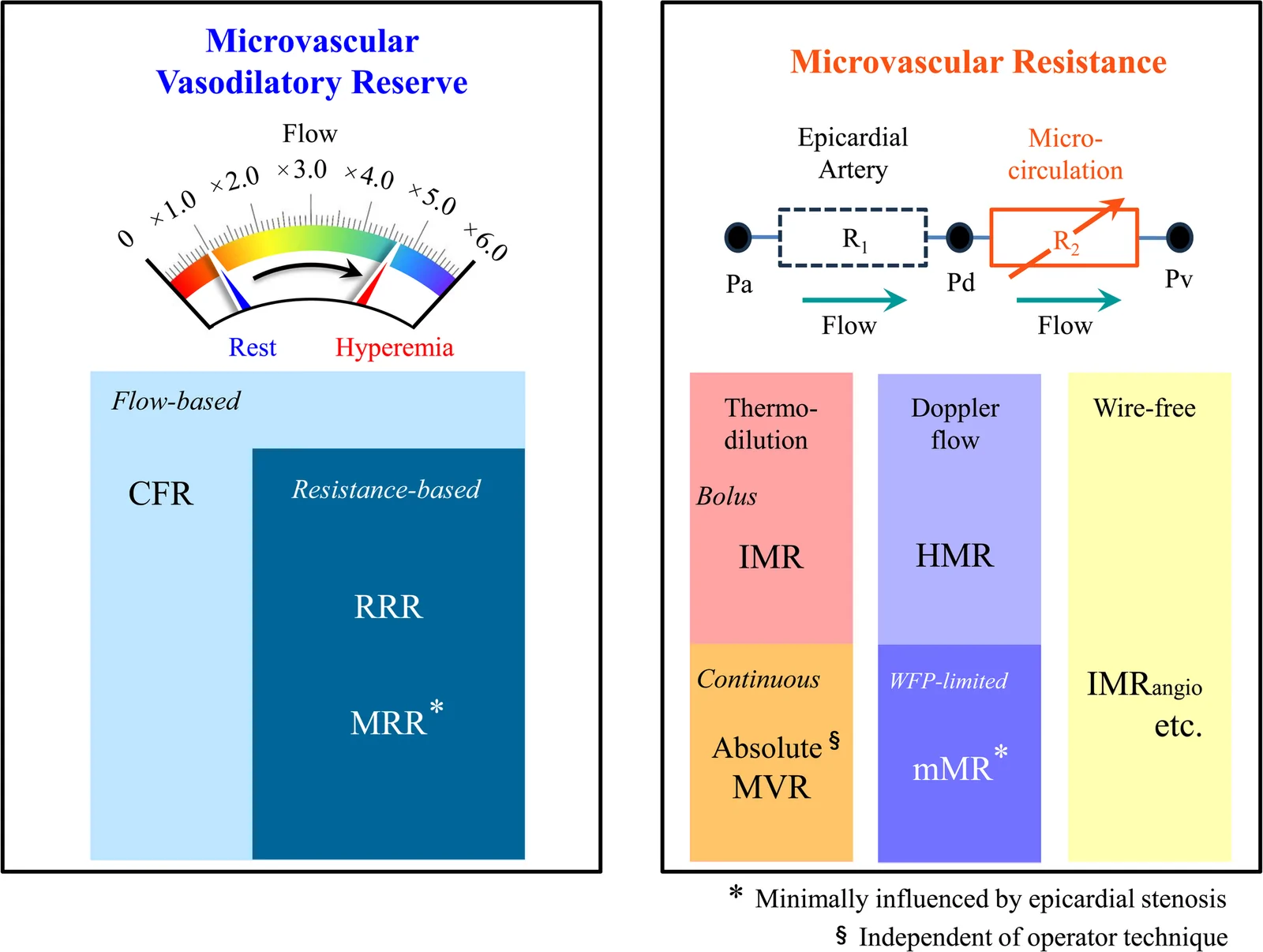
Overview of Current Invasive Physiological Indices for CMD. Physiological indices for coronary microvascular dysfunction (CMD) are organized into two categories: *vasodilatory reserve* (flow-based: CFR; resistance-based: RRR, MRR) and *microvascular resistance* (IMR, HMR, mMR, IMR_angio_ ​, absolute MVR). Symbols (*, §) indicate specific index characteristics: * denotes indices minimally influenced by epicardial stenosis, and § denotes independence from operator technique

Several physiological indices are currently available for evaluating coronary microvascular function in clinical practice [17, 25]. These indices are broadly categorized into two major groups: those assessing microvascular vasodilatory reserve capacity and those quantifying MVR. Each index provides an independent assessment of CMD, yet they capture different aspects of microvascular function and are complementary in offering deeper insight and improving risk stratification, as discussed later [33–38]. In the following section, we briefly describe the physiological background of each index and review key evidence supporting their clinical relevance. In interpreting these indices, it should be noted that currently used diagnostic cut-off values for vasodilatory reserve and microvascular resistance should be applied with caution, as these physiological parameters are continuous variables and are influenced by multiple hemodynamic, methodological, and patient-related factors; therefore, careful clinical judgment would be required rather than strict dichotomization. Figure 2 provides an overview of current physiological indices, categorized by vasodilatory reserve and microvascular resistance, while Table 1 summarizes their definitions, strengths, and limitations.

**Table 1:** Summary of intracoronary physiological indices for the assessment of coronary microvascular dysfunction

Type of Index	Index	Flow Assessment Method	Calculation Formula	Threshold	Strengths	Limitations
**Microvascular Vasodilatory Reserve**						
Flow-based	CFR	All modalities	Flow_hyper/Flow_rest	<2.0–2.5	Widely validated across multiple modalities.	Cannot distinguish microvascular dysfunction from epicardial involvement
					Demonstrates prognostic relevance in multiple clinical scenarios, including INOCA and heart failure.	Influenced by systemic hemodynamic conditions
					Supported by the strongest and most consistent body of evidence among current indices.	Inter-method variability; Doppler-based CFR offers better accuracy, whereas bolus thermodilution-based CFR is more feasible
						Prognostic utility may be limited in ACS
MVR-based	RRR	All modalities	MVR_rest/MVR_hyper = CFR × Pd_rest/Pd_hyper	<2.6–3.5	Quantifies microvascular vasodilatory reserve more specifically by integrating flow and pressure	Limited clinical validation and insufficient prognostic evidence
					Less influenced by systemic hemodynamic fluctuations compared to CFR	Lack of consensus on optimal cut-off values
					Relatively easy to measure in clinical settings	Not yet widely adopted in routine clinical practice
	MRR	All modalities	(Flow_hyper/Flow_rest) × (Pa_rest/Pd_hyper) = (CFR/FFR) × (Pa_rest/Pa_hyper)	<2.5–3.0	Specifically designed to assess microvascular vasodilatory capacity independently of epicardial disease	Based on physiological assumptions that may not hold in all patients
					May provide better prognostic utility than CFR, particularly in patients with CAD	Cut-off values are not yet standardized or widely validated
					Developed to be applicable across various invasive modalities	
**Microvascular Resistance Indices**						
Intracoronary flow & pressure-based	IMR	Bolus thermodilution	IMR = Pd × Tmn during hyperemia	≥25U (INOCA/CCS)	Provides a specific, quantitative assessment of coronary microvascular resistance	May be overestimated in the presence of significant epicardial stenosis without correction; correction using Pw remains debated
			If there is a functionally coronary stenosis:		Less affected by epicardial stenosis and systemic hemodynamics compared to CFR	Cut-off values may vary across clinical contexts; optimal thresholds not yet standardized
			IMR (Pw-corrected) = Pa × Tmn × [(Pd − Pw)/(Pa − Pw)]	≥40U (STEMI)	Validated as a prognostic marker in STEMI	Assessment location may affect its values
			IMR(Yong’s formula)= Pa × Tmn × ([1.35 × Pd/Pa] − 0.32)		More feasible in clinical practice compared to HMR	Limited prognostic utility outside of STEMI
	HMR	Doppler flow	HMR = Pd/APV during hyperemia	≥2.5 mmHg/cm/sec	Directly quantifies microvascular resistance using Doppler flow velocity	Dependent on high-quality Doppler signals, which are technically demanding
					Less influenced by downstream myocardial mass compared to IMR	Lower feasibility compared to thermodilution-based methods
					More sensitive to myocardial injury than IMR, with better prognostic value in STEMI	May still be affected by myocardial mass in small perfusion territories
					Applicable even with moderate epicardial stenosis (FFR >0.60)	Optimal cut-off value remains uncertain, limiting widespread clinical adoption
						Impact of collateral flow remains controversial, even when FFR >0.60
	mMR	Doppler flow	mMR = Pd/APV during the wave-free period and hyperemia		Assesses microvascular resistance during the wave-free period, minimizing systolic influence	Requires high-quality Doppler signals and specialized software for diastolic phase analysis
					Offers a more specific evaluation of intrinsic microvascular resistance with minimal influence from epicardial stenosis	Technically demanding, with limited feasibility in routine clinical settings
						Prognostic value remains to be validated
	Absolute MVR	Continuous thermodilution	Absolute MVR = Pd/Absolute coronary flow during hyperemia		Provides an absolute value of microvascular resistance (mmHg/L/min, Wood units)	Requires a dedicated infusion catheter
					Operator-independent technique with high reproducibility	Not yet integrated into routine clinical workflows
					Validated against PET/CT-derived MVR	Optimal cut-off values have not been established
					Does not require pharmacologic hyperemia due to infusion-induced steady vasodilation	May be influenced by assessment location and the presence of epicardial stenosis
						Prognostic value remains to be clarified
Angio-based	IMR_angio_	Frame counts	IMR_angio_= Pa × QFR × Nframes/fps		Wire-free, angiography-derived method for assessing MVR	Flow estimation is based on frame count analysis, which may lack accuracy
					Demonstrates acceptable correlation with wire-based IMR across various clinical settings (e.g., STEMI, NSTE-ACS, CCS)	Agreement with wire-based IMR is limited and varies across clinical settings
					Particularly useful in acute settings (e.g., STEMI) where wire-based assessment is challenging	Prognostic value and definitive clinical role remain to be established
					Cost-effective and widely applicable in routine coronary angiography	

#### Coronary flow reserve (CFR)

##### Concept

**Fig. 3:**
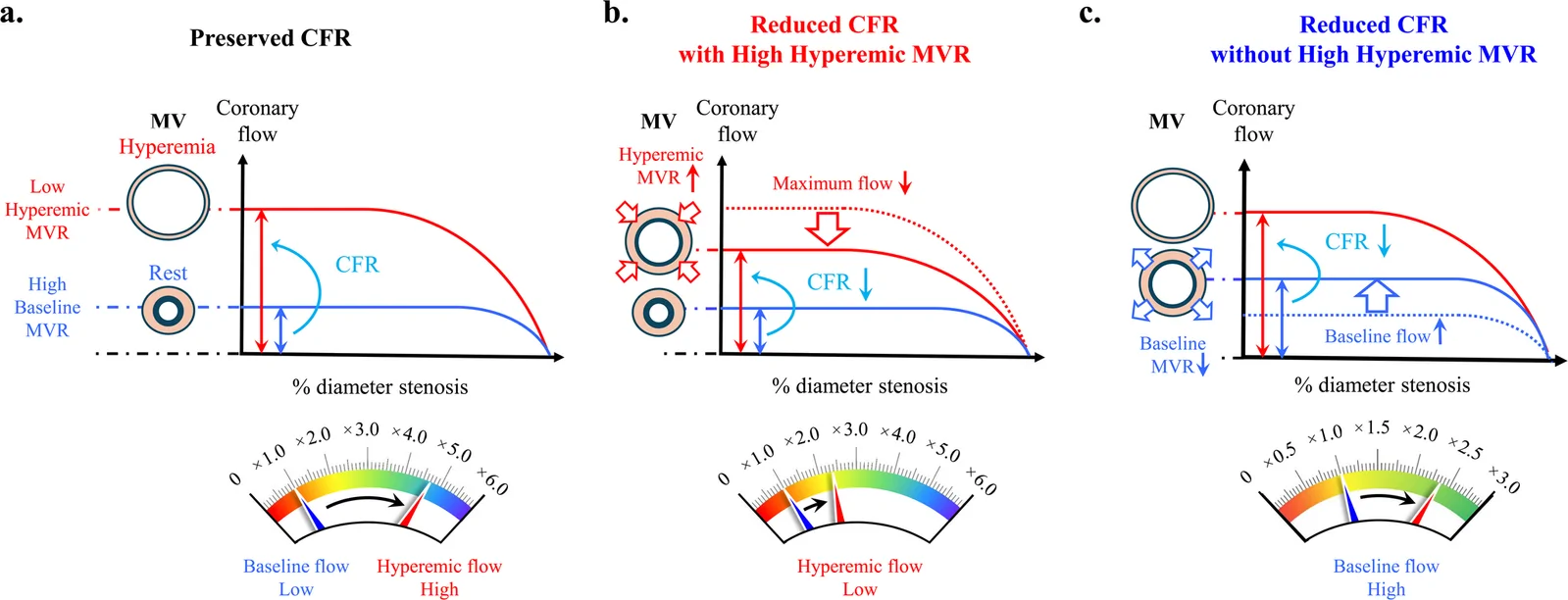
hysiological Concept of CFR and MVR-Based Classification of CMD Endotypes. Panel a illustrates the physiological principle of coronary flow reserve (CFR). Panels b and c demonstrate distinct CMD endotypes classified according to the presence or absence of elevated microvascular resistance (MVR). The x-axis represents percent diameter stenosis of the coronary artery, and the y-axis coronary flow. “MV” denotes the microvasculature

CFR represents the capacity of the coronary circulation to augment blood flow in response to increased myocardial metabolic demand, primarily through microvascular vasodilation [13, 39]. The concept was introduced in the 1970 s based on PET studies, initially to assess myocardial ischemia caused by epicardial coronary stenosis [40]. CFR is defined as the ratio of coronary flow under maximal vasodilation to baseline flow, representing the microvascular dilatory reserve capacity (Fig. 3a). CFR can be reduced through the following mechanisms, which may coexist and interact:

- **Mechanisms related to epicardial artery stenosis**:
  - Reduced maximal hyperemic flow due to epicardial narrowing.
  - Compensatory microvascular vasodilation at rest to maintain distal perfusion.
- **Mechanisms related to coronary microvascular conditions**:
  - Elevated resting coronary flow due to various systemic, cardiac, or microcirculatory conditions.
  - Increased MVR during hyperemia, limiting maximal coronary flow (e.g., inward remodeling, rarefaction, extramural compression).

Therefore, CFR reflects the integrated function of the entire coronary circulation. In contemporary practice, it has become a crucial index for evaluating coronary microcirculation, if functionally significant epicardial stenosis is excluded.

##### Definition/calculation

CFR can be assessed using either Doppler-derived flow velocity or thermodilution-based techniques, as well as non-invasive myocardial perfusion imaging [17, 25]. The invasive methods of calculating CFR, each corresponding to a specific flow measurement technique, are summarized below:

- Doppler flow velocity-based CFR = APV_hyper_/APV_rest_.
- Bolus thermodilution-based CFR = Tmn_rest_/Tmn_hyper_.
- Continuous thermodilution-based CFR = Q_hyper_/Q_rest_.

In the equations above, thesubscripts ‘rest’ and ‘hyper’ denote resting and hyperemic conditions, respectively.

Under normal physiological conditions, CFR typically ranges from 3.0 to 5.0. A reduced CFR is commonly defined by cut-off values of < 2.0 or < 2.5, depending on the measurement modality and clinical context [41]. 

##### Clinical relevance

CFR is a well-established physiological index with strong prognostic significance across a broad spectrum of cardiovascular diseases, such as chronic coronary syndrome (CCS), heart failure, and myocardial diseases [10, 42]. A meta-analysis showed that abnormal CFR is significantly associated with increased risk of mortality (HR: 3.78, 95% CI: 2.39–5.97) and major adverse cardiovascular events (HR: 3.42, 95% CI: 2.92–3.99) [42]. The association between CFR and adverse events holds true across different assessment modalities, both non-invasive and invasive. However, CFR values may vary depending on the measurement method. CFR derived from the bolus thermodilution technique moderately correlates with　CFR derived from Doppler velocity (*r* = 0.60), but tends to overestimate it, with a mean bias of 0.59 ± 1.24 [43]. This difference increases as CFR values rise. When PET-based CFR is used as the reference, CFR derived from Doppler velocity shows a stronger correlation (*r* = 0.82) than CFR derived from bolus thermodilution (*r* = 0.55) [44]. Meanwhile, CFR derived from continuous thermodilution has demonstrated excellent agreement with CFR derived from Doppler velocity (*r* = 0.90), with minimal bias and no systematic or proportional error [30]. It is therefore considered a reliable alternative. The concept of CFR has evolved into a new generation of resistance-based microvascular vasodilatory reserve indices, such as the Resistive Reserve Ratio (RRR) and Microvascular Resistance Reserve (MRR), which will be discussed in later sections.

#### Microvascular resistance indices

**Fig. 4:**
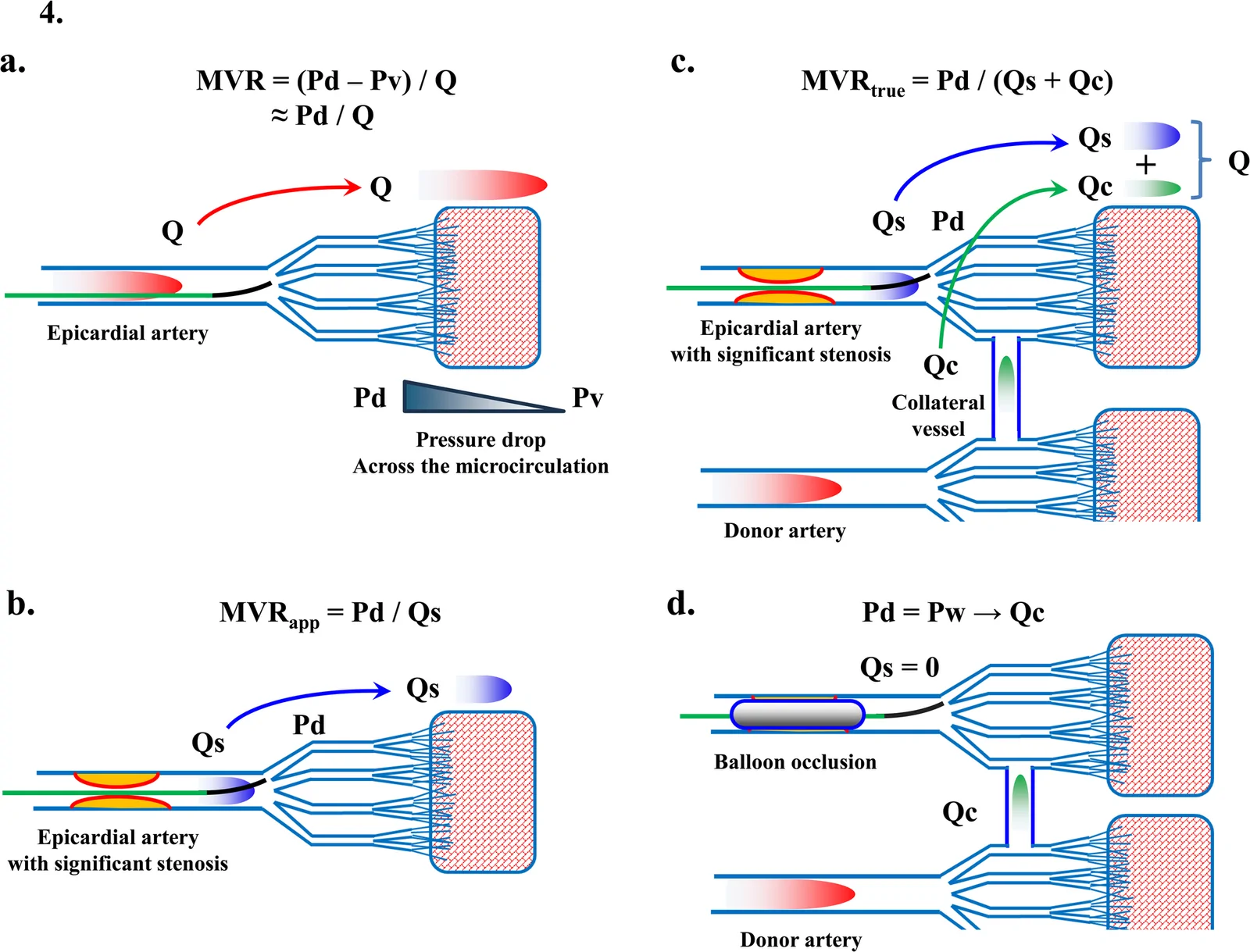
Principles of microvascular resistance assessment and its limitation under significant epicardial stenosis. In the absence of significant epicardial stenosis (Panel a), coronary flow measured by the sensor wire (Q) represents the total myocardial flow within the target territory, and microvascular resistance can be expressed simply as Pd/Q, assuming that central venous pressure (Pv) is negligible compared with distal pressure (Pd). When a severe epicardial stenosis is present (Panels b–d), collateral flow (Qc) partially maintains myocardial perfusion distal to the stenosis, whereas antegrade flow through the stenotic segment (Qs) decreases. In this setting, distal coronary pressure (Pd) is supported by collateral inflow. Consequently, the apparent microvascular resistance, calculated simply as Pd/Qs (MVR_app_), becomes higher than the true value, defined as Pd/(Qs + Qc) (MVR_true_), leading to an overestimation of microvascular resistance. To evaluate the contribution of collateral flow (Qc), coronary wedge pressure (Pw) obtained during balloon occlusion can be used; however, its interpretation remains a matter of debate

The concept of hyperemic MVR indices has been introduced as a quantitative approach to assess the minimal achievable resistance within the coronary microcirculation [21, 45]. Based on Ohm’s law, hyperemic MVR indices are defined as the ratio of the pressure drop across the coronary microcirculation to coronary flow during maximal vasodilation (Fig. 4a):

$$\mathbf{M}\mathbf{V}\mathbf{R}=(\mathbf{P}\mathbf{d}-\mathbf{P}\mathbf{v})/\mathbf{F}\mathbf{l}\mathbf{o}\mathbf{w}\hspace{0.17em}\approx\hspace{0.17em}\mathbf{P}\mathbf{d}/\mathbf{F}\mathbf{l}\mathbf{o}\mathbf{w}$$

Where Pv denotes central venous pressure. Since Pv is generally negligible compared with Pd, MVR is simply approximated as Pd divided by flow [46]. These indices are derived solely from the pressure–flow relationship during hyperemia. Therefore, unlike CFR, which is influenced by resting vascular tone and systemic hemodynamics, MVR indices are less susceptible to physiological variability, although recent pathophysiological studies have shown that distinct information is withheld in measurements during the resting state [47]. A higher hyperemic MVR indicates greater impairment in myocardial flow augmentation. Moreover, MVR indices are considered to better reflect structural abnormalities of the coronary microcirculation [17, 48–52]. Although the prognostic and diagnostic evidence for MVR is relatively limited in patients with CCS, it is more robust in the context of acute coronary syndromes (ACS), where elevated MVR has been associated with impaired microvascular reperfusion and worse clinical outcomes. The measurements of MVR are generally performed with the sensor positioned about two-thirds down the coronary artery. It should be noted, however, that MVR values may vary depending on the measurement site, even within the same vessel. This can be partially explained by the fact that in anatomically normal arteries, distal coronary perfusion pressure remains relatively stable, whereas coronary flow declines toward the distal segments [53]. As a result, MVR tends to appear higher in more distal locations [54, 55]. These methodological variations highlight the need for further standardization and validation of measurement protocols in future studies.

#### Index of microcirculatory resistance (IMR)

##### Concept/background

IMR was introduced in 2003, shortly after the development of bolus thermodilution-based flow estimation [45]. IMR quantifies the minimal MVR during maximal hyperemia, and its validity has been supported by a significant correlation with true MVR, as measured using an external flow probe in open-chest porcine models (*R* = 0.53, *P* < 0.0001). This index is considered less affected by epicardial disease and systemic hemodynamics leading to higher reproducibility compared with CFR [47, 56]. 

##### Definition/calculation

IMR is defined as the product of Pd and Tmn during maximal hyperemia:

$$\mathbf{I}\mathbf{M}\mathbf{R}=\mathbf{P}\mathbf{d}\times\mathbf{T}\mathbf{m}\mathbf{n}\,\,\mathbf{d}\mathbf{u}\mathbf{r}\mathbf{i}\mathbf{n}\mathbf{g}\,\,\mathbf{h}\mathbf{y}\mathbf{p}\mathbf{e}\mathbf{r}\mathbf{e}\mathbf{m}\mathbf{i}\mathbf{a}$$

In the presence of significant epicardial stenosis, collateral flow may lead to an overestimation of IMR (Fig. 4b and c). To account for this, wedge pressure (Pw), obtained during balloon occlusion, is used to correct for collateral contribution (Fig. 4d):

$$\mathbf{IMR(Pw-corrected) \\ = Pa \times Tmn \times [(Pd - Pw)/(Pa - Pw)]}$$

where Pa represents aortic pressure. Alternatively, IMR can be estimated without balloon occlusion using a simplified formula as follows [57]

$$\mathbf{IMR(Yong's \ formula) \\ = Pa \times Tmn \times ([1.35 \times Pd / Pa] - 0.32)}$$

This method enables calculation even when epicardial stenosis is present. However, the validity of Pw correction in clinical settings remains a matter of debate.

##### Clinical relevance

The prognostic significance of IMR was first established in the context of ST-segment elevation myocardial infarction (STEMI). Higher IMR values after primary PCI were associated with larger infarct size, reduced wall motion recovery, and the presence of microvascular obstruction on MRI [49, 51]. A prospective multicenter study involving 253 STEMI patients demonstrated that IMR > 40 U predicted higher rates of mortality and heart failure-related rehospitalization (20.0% vs. 11.0%, *P* = 0.04) [48]. In multivariate analysis, IMR > 40 U was the only independent predictor of death (HR 4.34, 95% CI 1.26–15.00; *P* = 0.02). Based on these accumulated data, IMR provides a quantitative assessment of myocardial injury and serves as an important prognostic marker in the setting of STEMI. In non-ST elevation acute coronary syndrome (NSTE-ACS), the evidence for prognostic utility is limited. A small retrospective study showed worse outcomes in patients with higher IMR (≥ 22.9, χ^2^ = 7.12; *P* = 0.008) and identified IMR as an independent predictor of MACE [58]. However, the main adverse events were driven by repeated revascularization which differs from the STEMI setting.

In patients with CCS or those without obstructive CAD, the prognostic value of IMR alone remains uncertain. An IMR value of ≥ 25 U has been suggested as an abnormal threshold in this population; however, previous studies in CCS patients have reported lower cutoff values for predicting clinical events, suggesting that the prognostic utility of this threshold warrants further evaluation [35, 59, 60]. Furthermore, IMR is primarily regarded as a complementary tool to CFR, although current guidelines recognize IMR alone as a diagnostic marker of CMD [1, 61]. This will be further discussed in the next section on clinical presentation.

#### Hyperemic microvascular resistance (HMR)

##### Concept/background

HMR is a Doppler flow velocity-based index of MVR, introduced in 2001 [21]. It shares a conceptual basis with IMR, aiming to quantify MVR during maximal hyperemia. Initially, HMR was used as a specific marker of microvascular function, particularly for the interpretation of CFR and FFR [21, 62]. 

##### Definition/calculation

HMR is defined as the ratio of Pd to APV during hyperemia:

$$\mathbf{H}\mathbf{M}\mathbf{R}\hspace{0.17em}=\hspace{0.17em}\mathbf{P}\mathbf{d}/\mathbf{A}\mathbf{P}\mathbf{V}\,\,\mathbf{d}\mathbf{u}\mathbf{r}\mathbf{i}\mathbf{n}\mathbf{g}\,\,\mathbf{h}\mathbf{y}\mathbf{p}\mathbf{e}\mathbf{r}\mathbf{e}\mathbf{m}\mathbf{i}\mathbf{a}$$

Unlike flow volume-based indices such as IMR, HMR is derived from flow velocity, which remains relatively stable across coronary bifurcations [53, 54]. This makes HMR less affected by measurement location and downstream myocardial mass. However, even HMR may still be influenced by myocardial mass, particularly in vessels supplying smaller perfusion territories [55]. HMR can be measured across a wide range of epicardial stenosis severity and is generally considered reliable when FFR is above 0.60, as the influence of collateral flow is assumed to be negligible [46]. However, the influence of collateral circulation on HMR measurements—even when FFR is above 0.60—remains a matter of debate.

##### Clinical relevance

HMR, similar to IMR, is considered to reflect structural microvascular alterations such as capillary rarefaction [63]. Although both indices are derived from the same fundamental principle of pressure divided by flow during hyperemia, the correlation between HMR and IMR is only modest (rho = 0.39, *p* = 0.0006), suggesting that they may differ in predictive performance [64]. In the setting of STEMI, HMR was more sensitive to myocardial injury compared to IMR, and elevated HMR was also associated with adverse outcomes [64–66]. Regarding its predictive utility, in patients with non-obstructive CAD, an HMR ≥ 1.9 mmHg/cm/sec has been associated with recurrent chest pain, and a threshold of ≥ 2.5 mmHg/cm/sec has been proposed to delineate elevated MVR, since this threshold reflected the upper limit of normal in patients with non-cardiac chest pain [50, 65, 67]. 

#### Minimal microvascular resistance (mMR)

##### Concept/background

mMR is a Doppler-derived index proposed to assess MVR during the hyperemic wave-free period in diastole [68]. This phase is characterized by minimized and stable MVR, due to the absence of systolic myocardial compression. mMR was introduced as a novel method to overcome the limitations of conventional hyperemic indices that may be affected by systolic variability or epicardial disease.

##### Definition/calculation

mMR is calculated as the ratio of Pd to APV measured specifically during the wave-free period under hyperemia.

$$\begin{aligned}&\mathbf{m}\mathbf{M}\mathbf{R}\hspace{0.17em}=\mathbf{P}\mathbf{d}/\mathbf{A}\mathbf{P}\mathbf{V}\,\,\\&\hspace{0.17em}\mathbf{d}\mathbf{u}\mathbf{r}\mathbf{i}\mathbf{n}\mathbf{g}\,\,\mathbf{t}\mathbf{h}\mathbf{e}\,\mathbf{w}\mathbf{a}\mathbf{v}\mathbf{e}-\mathbf{f}\mathbf{r}\mathbf{e}\mathbf{e}\,\,\mathbf{p}\mathbf{e}\mathbf{r}\mathbf{i}\mathbf{o}\mathbf{d}\,\,\mathbf{a}\mathbf{n}\mathbf{d}\,\,\mathbf{h}\mathbf{y}\mathbf{p}\mathbf{e}\mathbf{r}\mathbf{e}\mathbf{m}\mathbf{i}\mathbf{a}\end{aligned}$$

This diastolic timing is intended to reduce the influence of intramyocardial pressure as well as collateral flow, which are more prominent during systole [69]. 

##### Clinical relevance

mMR may offer a more selective evaluation of MVR independent of epicardial disease [68]. Unlike conventional indices such as CFR or HMR, mMR shows minimal dependence on epicardial stenosis severity, as evidenced by its lack of significant correlation with FFR (*r* = 0.055, *P* = 0.32), in contrast to CFR (*r* = 0.488, *P* < 0.001) and HMR (*r* = − 0.159, *P* = 0.03). However, its clinical use remains limited due to the need for specialized offline analysis software and technical complexity. Further validation is required to establish its prognostic value and potential role in routine practice.

#### Absolute coronary flow-based microvascular resistance (Absolute MVR)

##### Concept/background

Absolute MVR is a novel index for assessing coronary MVR using continuous thermodilution-derived coronary flow [70]. It was developed to provide a more direct and operator-independent measurement of MVR for overcoming the limitations of Doppler velocity or bolus thermodilution methods.

##### Definition/calculation

Absolute MVR is defined as the ratio of Pd to absolute coronary flow during hyperemia, expressed in mmHg/L/min (Wood units):

$$\mathbf{A}\mathbf{b}\mathbf{s}\mathbf{o}\mathbf{l}\mathbf{u}\mathbf{t}\mathbf{e}\,\,\mathbf{M}\mathbf{V}\mathbf{R}\hspace{0.17em}=\hspace{0.17em}\mathbf{P}\mathbf{d}/\mathbf{Q}\,\mathbf{d}\mathbf{u}\mathbf{r}\mathbf{i}\mathbf{n}\mathbf{g}\,\,\mathbf{h}\mathbf{y}\mathbf{p}\mathbf{e}\mathbf{r}\mathbf{e}\mathbf{m}\mathbf{i}\mathbf{a}$$

Hyperemic coronary flow is determined by continuous intracoronary saline infusion at a rate of 15–25 mL/min using a dedicated infusion catheter, with infusion speeds adapted to the coronary anatomy [30]. This technique does not require pharmacologic hyperemia, as the infusion of saline itself through a dedicated infusion catheter (RayFlow, Hexacath, France) induces steady-state maximal vasodilation, most likely through intravascular hemolysis [32]. 

##### Clinical relevance

Absolute MVR has been validated against PET-derived MVR [29]. Its reproducibility and independence from operator technique are key advantages [70]. However, like traditional MVR indices, Absolute MVR can be influenced by the assessment location as well as epicardial stenosis [71]. Furthermore, the need for a dedicated infusion catheter and the lack of a standardized cut-off value currently limit its utility in clinical practice. Further studies are required to clarify its prognostic value and establish reference ranges.

#### Angiography-derived index of microcirculatory resistance (IMR_angio_)

##### Concept/background

IMR_angio_ is a wire-free, angiography-derived index for assessing coronary MVR, first introduced in 2020 [72, 73]. It was developed to facilitate physiological assessment without the need of intracoronary sensor-equipped wires, offering a practical alternative in situations when conventional assessments are not feasible. A key feature of this method is the use of quantitative flow ratio (QFR), derived from computational fluid dynamics computed using 3D coronary angiographic modeling, originally developed as a surrogate for FFR.

##### Definition/calculation

IMR_angio_ is calculated in the same manner as conventional IMR equation, using angiography-based surrogates for both pressure and flow:

- Pd is estimated as Pa × QFR.

where QFR is the angiographically derived estimate of the hyperemic Pd/Pa ratio.

- Hyperemic flow is approximated using frame count analysis:
- N_frames_/fps, where N_frames_ is the number of angiographic frames required for contrast to travel from the guiding catheter to the distal vessel, and fps is the acquisition frame rate.

Thus, the IMR_angio_ formula becomes:

$$\mathbf{I}\mathbf{M}\mathbf{R}_\mathbf{angio}=\mathbf{P}\mathbf{a}\times\mathbf{Q}\mathbf{F}\mathbf{R}\times\mathbf{N}_\mathbf{frames}/\mathbf{f}\mathbf{p}\mathbf{s}$$

##### Clinical Relevance

IMR_angio_ was initially validated in STEMI patients, where pressure-wire-based IMR assessment is often clinically challenging [73]. In this setting, it showed a strong correlation with wire-based IMR (ρ = 0.85, *p* < 0.001) and high diagnostic accuracy for predicting post-PCI IMR > 40U (AUC = 0.96) and microvascular obstruction (AUC = 0.81). In NSTE-ACS and CCS, post-PCI IMR_angio_ remained significantly correlated with IMR (NSTE-ACS: ρ = 0.72; CCS: ρ = 0.70; both *p* < 0.001) [74]. In a subgroup of CCS patients without obstructive CAD (FFR > 0.80), IMR_angio_ values were significantly higher in patients with high IMR (> 25U) compared to those without high IMR (31.0 [25.3–42.5] vs. 16.6 [14.1–19.7], *p* < 0.001), suggesting its potential utility in identifying microvascular angina. IMR_angio_ may facilitate the assessment of coronary microcirculation in clinical practice as a convenient and cost-effective tool. In recent years, several wire-free methods for assessing MVR, like this index, have been proposed [72]. However, despite its potential as a wire-free alternative, notable differences remain between IMR_angio_ and wire-based IMR. The limits of agreement vary considerably across clinical settings—45U for STEMI, 21U for NSTE-ACS, and 15U for CCS—raising concerns about its measurement accuracy and underscoring the need for further validation [74]. 

#### Indices of resistance-based microvascular vasodilatory reserve

**Fig. 5:**
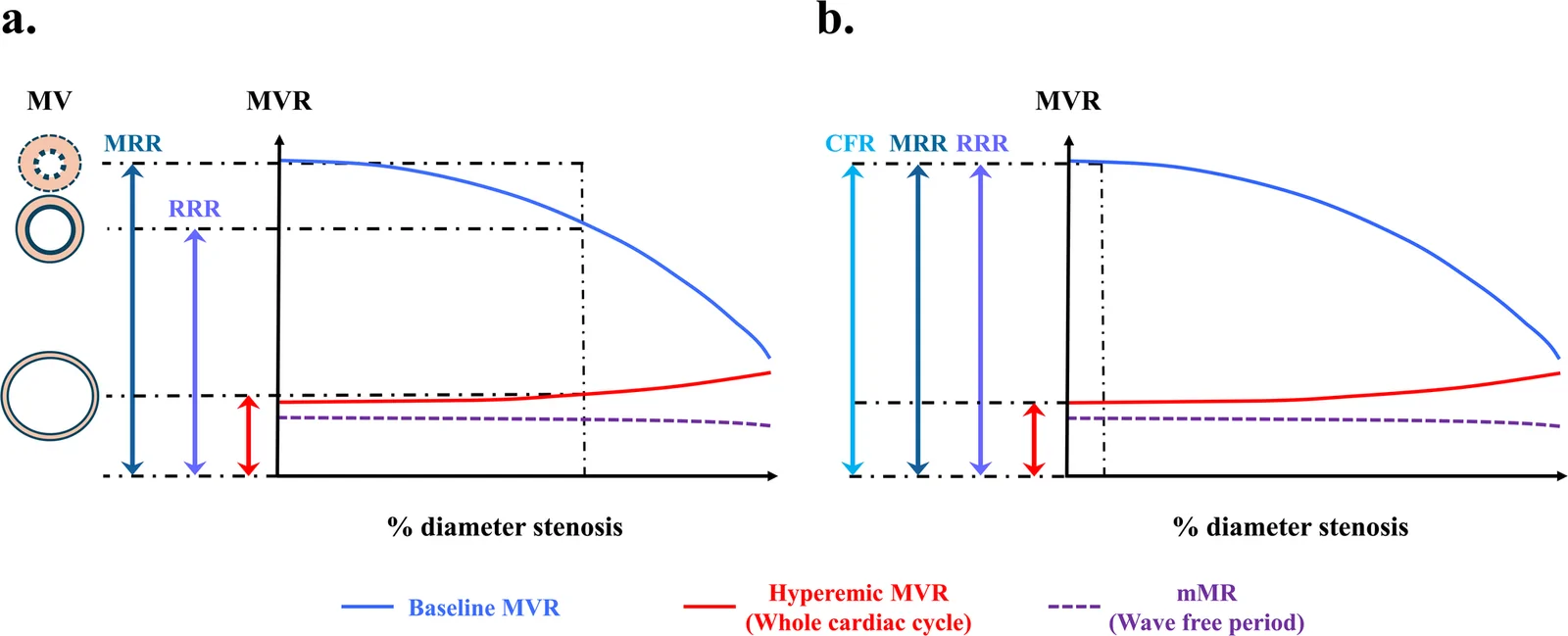
Concept of Novel Physiological Indices for CMD evaluation. This figure illustrates the conceptual evolution of physiological indices for CMD assessment, including RRR, MRR, and mMR, in relation to epicardial stenosis and MVR. Panel A shows the relationship between each index and MVR across varying degrees of stenosis. RRR and MRR quantify the dynamic change in MVR between resting and hyperemic conditions, whereas mMR represents the minimal achievable MVR specifically measured during the wave-free period of diastole under hyperemia. In an idealized condition without epicardial stenosis and with stable systemic pressure following hyperemia (Panel b), RRR and MRR theoretically become equivalent to CFR, as all indices reflect the same vasodilatory capacity of the microcirculation. MRR and mMR are considered to be less influenced by epicardial stenosis, representing indices that more selectively evaluate the microcirculation. Figure conceptually modified from de Waard GA, et al., Eur Heart J. 2016;37:2069–2080 and Eur Heart J. 2018;39:4062–4071

The concept of resistance-based microvascular vasodilator reserve, represented by indices such as RRR and MRR, builds upon the principles of CFR. These indices are specifically designed to quantify the change in MVR from resting to maximal vasodilation, and are derived from combined assessments of coronary pressure and flow. Figure 5 illustrates the conceptual basis of RRR and MRR in relation to epicardial stenosis and microvascular resistance.

#### Resistive reserve ratio (RRR)

##### Concept/background

RRR is an integrated physiological index based on coronary flow and pressure that evaluates the change in MVR from rest to hyperemia [75]. It represents a pressure-adjusted form CFR, enabling a more direct assessment of microvascular function by accounting for changes in driving pressure. It was first introduced in 2013 and has gained renewed attention since 2020 [76, 77]. 

##### Definition/calculation

RRR is defined as the ratio of baseline MVR to hyper MVR:

$$\begin{array}{*{20}{l}} {{\bf{RRR}}{\rm{ }} = {\rm{ }}{\bf{baseline}}{\rm{ }}\,\,{\bf{MVR}}{\rm{ }}/{\rm{ }}{\bf{hyper}}\,\,{\rm{ }}{\bf{MVR}}} \\ { = \hspace{0.17em} {\rm{ }}\left( {{\bf{P}}{{\bf{d}}_{{\bf{rest}}}}/{\rm{ }}{\bf{Flo}}{{\bf{w}}_{{\bf{rest}}}}} \right){\rm{ }}/{\rm{ }}\left( {{\bf{P}}{{\bf{d}}_{{\bf{hyper}}}}/{\rm{ }}{\bf{Flo}}{{\bf{w}}_{{\bf{hyper}}}}} \right)} \\ \hspace{0.17em} { = {\rm{ }}{\bf{P}}{{\bf{d}}_{{\bf{rest}}}}/{\bf{P}}{{\bf{d}}_{{\bf{hyper}}}} \times {\rm{ }}{\bf{CFR}}} \end{array}$$

By incorporating both pressure and flow at two physiological states, RRR quantitatively reflects the vasodilator reserve of the coronary microcirculation. This concept can be applied across different flow assessment modalities, including Doppler and thermodilution.

##### Clinical relevance

While only a limited number of studies have evaluated its clinical role, RRR has been investigated across various disease settings. In STEMI, lower RRR values have been associated with larger infarct size and greater microvascular obstruction. An RRR ≤ 1.6 was identified as a threshold for predicting heart failure or death (AUC = 0.62, *p* = 0.040), though it did not show superior prognostic value over CFR [77]. Conversely, in patients with CCS or non-obstructive coronary artery disease, RRR demonstrated better prognostic performance than CFR [76, 78]. However, reported optimal thresholds varied widely, from 2.62 to 3.5. This may be due to differences in flow assessment methods —Doppler flow in the former, and bolus thermodilution in the latter. Therefore, its clinical applicability remains to be standardized. Further research is needed to define its prognostic value and determine appropriate cut-off values across patient populations.

#### Microvascular resistance reserve (MRR)

##### Concept/background

MRR was introduced in 2021 as a novel physiological index to assess microvascular vasodilatory reserve, independently of epicardial coronary stenosis [31]. The concept was developed to overcome a key limitation of CFR, which can be confounded by compensatory microvascular vasodilation at rest to maintain adequate resting coronary flow. MRR seeks to eliminate the influence of autoregulatory compensation by conceptually comparing resting and hyperemic MVR under the assumption of a normal epicardial artery. Although its concept and calculation are relatively complex, the greater independence from epicardial stenosis may broaden the clinical applicability of microvascular assessment.

##### Definition/calculation

MRR is defined as the ratio of “true” resting MVR —assumed under a completely normal epicardial artery—to hyperemic MVR. The calculation is based on the following assumptions:

- In an ideally normal artery, Pa = Pd and resting coronary flow remains unchanged regardless of stenosis:

$$\begin{aligned}&{\rm{ }}{\bf{P}}{{\bf{a}}_{{\bf{rest}}}}={\rm{ }}{{\bf{Pd}}_{{\bf{rest}}\,{\rm{ }}\,{\bf{with}}\,{\rm{ }}{\bf{stenosis}}}}\end{aligned}$$

$${{\bf{Q}}_{{\bf{rest}}\,{\rm{ }}{\bf{with}}\,{\rm{ }}{\bf{stenosis}}}} = {\rm{ }}{{\bf{Q}}_{{\bf{rest}}\,{\rm{ }}\,{\bf{without}}\,{\rm{ }}{\bf{stenosis}}}}$$

- Resting MVR without stenosis is calculated as:

$$\begin{aligned}&{\bf{MV}}{{\bf{R}}_{{\bf{rest}}\,{\rm{ }}\,{\bf{without}}{\rm{ }}\,{\bf{stenosis}}}} \\&= {\rm{ }}{\bf{P}}{{\bf{d}}_{{\bf{rest}}\,{\rm{ }}\,{\bf{without}}\,{\rm{ }}{\bf{stenosis}}}}/{\rm{ }}{{\bf{Q}}_{{\bf{rest}}\,{\rm{ }}\,{\bf{without}}{\rm{ }}\,{\bf{stenosis}}}} \\&= {\rm{ }}{\bf{P}}{{\bf{a}}_{{\bf{rest}}}}/{\rm{ }}{{\bf{Q}}_{{\bf{rest}}\,{\rm{ }}\,{\bf{with}}\,{\rm{ }}{\bf{stenosis}}}}\end{aligned}$$

- Hyperemic MVR is assumed to be unaffected by epicardial stenosis:

$$\begin{aligned}&{\bf{MV}}{{\bf{R}}_{{\bf{hyper}}\,{\rm{ }}\,{\bf{without}}\,{\rm{ }}{\bf{stenosis}}}} \\&= {\rm{ }}{\bf{MV}}{{\bf{R}}_{{\bf{hyper}}\,{\rm{ }}\,{\bf{with}}\,{\rm{ }}{\bf{stenosis}}}}\; \\& = {\rm{ }}{\bf{P}}{{\bf{d}}_{{\bf{hyper}}}}/{\rm{ }}{{\bf{Q}}_{{\bf{hyper}}{\rm{ }}\,{\bf{with}}{\rm{ }}\,{\bf{stenosis}}}}\end{aligned}$$

Using these relationships, MRR can be derived as:

$$\begin{aligned}&{\bf{MRR}}{\rm{ }} \\&= {\rm{ }}{\bf{MV}}{{\bf{R}}_{{\bf{rest}}{\,}{\rm{ }}{\bf{without}}{\,}{\rm{ }}{\bf{stenosis}}}}/{\rm{ }}{\bf{MV}}{{\bf{R}}_{{\bf{hyper}}{\,}{\rm{ }}{\bf{without}}{\,}{\rm{ }}{\bf{stenosis}}}}\\& = {\rm{ }}\left( {{{\bf{Q}}_{{\bf{hyper}}{\rm{ }}{\,}{\bf{with}}{\rm{ }}{\,}{\bf{stenosis}}}}/{\rm{ }}{{\bf{Q}}_{{\bf{rest}}{\,}{\rm{ }}{\bf{with}}{\,}{\rm{ }}{\bf{stenosis}}}}} \right){\rm{ }} \\&\times {\rm{ }}\left( {{\bf{P}}{{\bf{a}}_{{\bf{rest}}}}/{\rm{ }}{\bf{P}}{{\bf{d}}_{{\bf{hyper}}}}} \right)\end{aligned}$$

This simplifies to:

$$\mathbf{MRR} = (\mathbf{CFR} / \mathbf{FFR}) \times (\mathbf{Pa}_{\mathrm{rest}} / \mathbf{Pa}_{\mathrm{hyper}})$$

Thus, MRR can be interpreted as a CFR adjusted by FFR and changes in aortic pressure between rest and hyperemia. In the ideal absence of epicardial stenosis (i.e. FFR = 1.00) and stable Pa during hyperemia, MRR equals CFR.

##### Clinical relevance

MRR was first validated using continuous thermodilution, showing a strong correlation with Doppler-derived MRR (*r* = 0.86, *p* < 0.001) [31]. Notably, CFR values declined with increasing epicardial stenosis severity (i.e., lower FFR), whereas MRR remained relatively stable, suggesting its independence from epicardial resistance. In a comparative study including data obtained from both Doppler and bolus thermodilution methods, MRR showed superior prognostic performance compared with CFR in patients with functional epicardial stenosis (FFR < 0.75) [79]. These findings suggest that MRR is less influenced by epicardial resistance, potentially overcoming the limitations of CFR in the presence of coexisting epicardial disease. Although MRR is based on several physiological assumptions—some of which are derived from idealized models of coronary physiology—it remains a promising index for the assessment of microvascular function. However, current evidence is limited to vessels with intermediate stenosis (FFR > 0.60). The diagnostic accuracy and prognostic relevance of MRR in more severe stenosis remain uncertain, as collateral flow is not explicitly accounted for in its calculation. Furthermore, reported optimal cutoff values for predicting adverse outcomes range from 2.5 to 3.0, but a definitive threshold has yet to be established [79–81]. Further validation studies are needed to clarify the clinical utility of MRR and to standardize its application across different patient populations.

## Clinical utility of invasive physiological assessments for coronary microcirculation

**Fig. 6:**
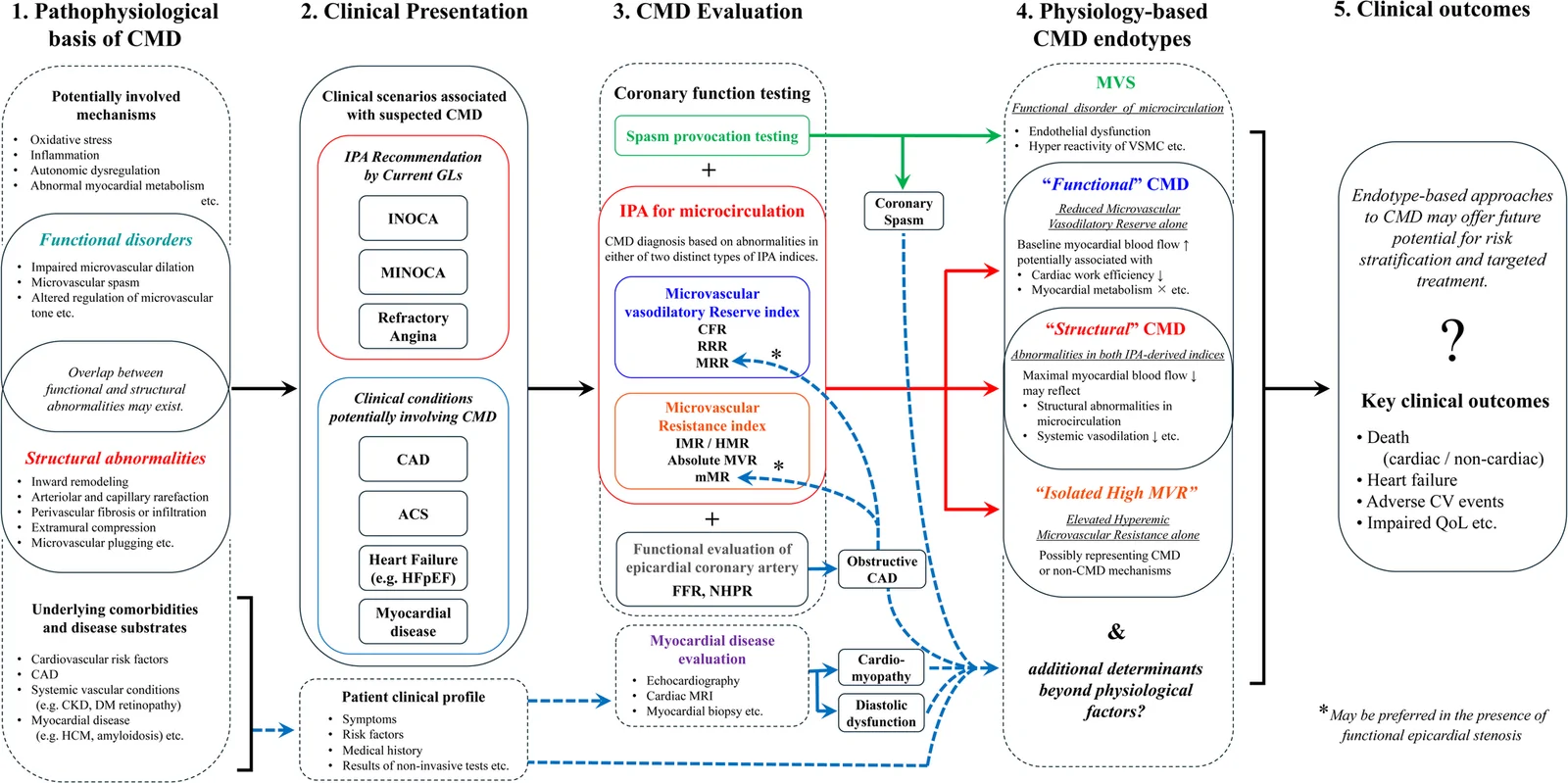
A Schematic Overview of CMD Evaluation: From Pathophysiological Basis to IPA-Based Endotyping. This figure presents an integrative conceptual framework for CMD evaluation, outlining the pathway from its pathophysiological basis to IPA-based CMD endotyping. It highlights the integration of coronary microvascular vasodilatory reserve and resistance indices to classify CMD into functional and structural endotypes, which may guide patient-specific management strategies. Furthermore, various clinical factors — such as underlying CAD, coronary spasm, myocardial disease, diabetic retinopathy, chronic kidney disease and diastolic dysfunction — may provide complementary information that further characterizes the CMD phenotype. In this schematic, dashed arrows represent conceptual flows that may be useful for a more detailed stratification of CMD across different clinical contexts, rather than definitive diagnostic pathways

As mentioned previously, the pathophysiological understanding of myocardial ischemia, including at the microvascular level, has significantly advanced, leading to growing clinical interest in CMD. IPA of the coronary microcirculation is now recognized as a valuable tool for elucidating the underlying mechanisms of ischemia and optimizing patient management, particularly in cases without significant epicardial stenosis [82]. Fig. 6 provides a schematic overview of CMD evaluation, outlining the flow from the pathophysiological basis of CMD to IPA-based endotyping and potential treatment considerations.

The CorMicA trial demonstrated that physiology-guided management, including vasoreactivity testing, led to greater improvements in symptoms and quality of life compared with conventional care [12]. This highlighted the importance of tailoring treatment strategies according to the underlying INOCA endotype—such as coronary spastic angina, CMD, or a combination of both—in order to achieve more accurate diagnosis, better risk stratification, and individualized therapy. Based on the findings of the CorMicA trial, this approach has been incorporated into clinical guidelines, underscoring that anatomical assessment alone—such as CT or coronary angiography—is insufficient to rule out ischemia in patients presenting with angina-like or unresolved chest symptoms [12]. Specifically, the 2019 ESC guidelines gave IPA a class IIa recommendation for patients with suspected INOCA who present with symptoms but without significant epicardial stenosis, and the 2024 update further strengthened this recommendation to class I, level B [1, 83]. More recently, the ILIAS ANOCA trial, published in 2025, confirmed and extended the findings of CorMicA, demonstrating that routine coronary function testing combined with tailored therapy significantly improved quality of life in patients with angina and no obstructive coronary arteries compared with standard care [84]. 

Given the wide spectrum of pathophysiological mechanisms and clinical presentations encompassed by CMD, some diagnostic criteria and endotype classifications have been proposed to support its clinical evaluation. Among these, the COVADIS group proposed standardized diagnostic criteria for MVA, consisting of the following four elements [41]:

1. Symptoms suggestive of myocardial ischemia;
2. Absence of obstructive coronary artery disease;
3. Objective evidence of myocardial ischemia;
4. Evidence of impaired coronary microvascular function.

The last criterion includes impaired vasodilator reserve (e.g., CFR ≤ 2.0–2.5.0.5), increased MVR (e.g., IMR > 25, HMR > 2.5mmHg/cm/s), and/or microvascular spasm. These standardized criteria serve as a foundation for the clinical evaluation of MVA, in which IPA plays a central role.

Based on clinical scenarios, Camici and Crea proposed a classification of CMD into four types [6]:

- Type 1: CMD in the absence of both myocardial disease and obstructive CAD;
- Type 2: CMD associated with myocardial disease;
- Type 3: CMD coexisting with obstructive CAD;
- Type 4: Iatrogenic CMD.

Type 1 CMD is considered a primary microvascular disorder and is representative of the INOCA population. Meanwhile, Type 2 CMD is associated with underlying myocardial diseases such as hypertrophic cardiomyopathy, cardiac amyloidosis, and Fabry disease. Identifying Type 2 CMD is clinically important, as these conditions carry distinct prognostic implications and require disease-specific management strategies [10]. However, current diagnostic algorithms for INOCA do not differentiate between Type 1 and Type 2 CMD [1]. As a result, patients initially diagnosed with Type 1 CMD (i.e., primary CMD) may later be reclassified as having Type 2 CMD when underlying myocardial disease becomes evident. To avoid such misclassification, clinicians should consider the possibility of myocardial disease in patients with abnormal ECG findings, left ventricular hypertrophy (LVH), or other distinctive clinical features. In such cases, additional investigations—such as cardiac MRI or myocardial biopsy—may be warranted. In this context, careful clinical evaluation beyond physiological assessment is essential to ensure accurate diagnosis and appropriate management, as CMD cannot be adequately characterized by catheterization findings alone, and its clinical significance may vary depending on symptom profiles, cardiovascular risk factors, medical history, and comorbidities. Specifically, among patients classified as having Type 1 CMD, coexisting clinical conditions (e.g., diastolic dysfunction) may influence risk profiles and subsequent management strategies. Accordingly, IPA is best interpreted in conjunction with relevant clinical information and non-invasive test results. (Fig. 6) The issue of Type 3 CMD, which refers to CMD in the presence of obstructive CAD, is discussed later in the section “Beyond INOCA”.

### A new framework to stratify CMD endotypes

CMD encompasses a wide spectrum of functional and structural abnormalities within the coronary microcirculation. In the CorMicA trial, CMD was identified in 72% of patients with suspected INOCA [85]. Among those with CMD, elevated hyperemic MVR, reduced CFR, and microvascular spasm were each observed in 45–48% of cases. Notably, around 35% of these patients exhibited two or more overlapping abnormalities, underscoring the heterogeneity and complexity of CMD endotypes. This finding suggests that the further endotypic classification of CMD may help guide more targeted treatment.

The microvascular dilatory reserve indices, represented by CFR, have demonstrated robust clinical relevance across a broad spectrum of cardiovascular diseases. As previously discussed, a reduced CFR can arise from different physiological endotypes—either with elevated hyperemic MVR or without. (Figure 3b and c) [86] Recently, based on these distinct physiological characteristics, Rahman and colleagues proposed a novel framework for stratifying CMD endotypes [33]. They classified CMD with reduced CFR and elevated hyperemic MVR as “structural” CMD, and CMD with reduced CFR but without elevated resistance as “functional” CMD. Importantly, the term “functional” in this context should be interpreted with caution, as it differs from the previously mentioned “pathophysiologically functional” CMD, which involves microvascular vasoconstriction or impaired vasodilation due to endothelial or smooth muscle dysfunction. Rahman highlighted distinct physiological profiles between "structural" and "functional" CMD .

“Structural” CMD is conceptually more straightforward, as limited maximal myocardial blood flow may result from structural alterations in the microvasculature. Their findings suggest that this endotype is associated with impaired systemic vasodilation, which, together with limited coronary flow augmentation due to elevated microvascular resistance, may synergistically contribute to exercise-induced myocardial ischemia [34]. 

In contrast, “functional” CMD is characterized by elevated resting coronary flow due to abnormally low baseline MVR. While high resting coronary flow can be seen in systemic conditions such as anemia or increased cardiac workload (e.g., hypertension), Rahman et al. observed that patients with “functional” CMD exhibited elevated resting coronary flow despite the absence of increased cardiac workload, which suggested an increase in resting myocardial oxygen demand and possibly reflected an inefficient myocardial metabolic state [34]. Previous studies have reported associations between elevated resting coronary flow and insulin resistance, as well as the presence of diabetic retinopathy—a known manifestation of systemic microvascular disease [87, 88]. Supporting this, van de Wouw et al. demonstrated in a metabolic swine model that such microcirculatory conditions were linked to reduced cardiac work efficiency and impaired myocardial metabolism [89]. These compromised myocardial and metabolic profiles may underlie what is referred to as “functional” CMD, suggesting a link to overall poorer clinical condition and adverse events.

Regarding the prognosis, some studies have reported that “structural” CMD is associated with a higher incidence of adverse cardiovascular events [35, 90]. However, Boerhout et al. found that the prognosis of both “structural” and “functional” CMD endotypes was comparable in terms of event rates [37]. A recent study from our group reported that the clinical factors associated with low baseline MVR, a physiological feature of “functional” CMD, differ completely from those associated with high IMR, and that this profile—closely resembling the risk factors for HFpEF—was linked to the occurrence of death and heart failure [91]. The prognostic relevance of this physiological endotype has been repeatedly reported [92–94]. These findings underscore the need for further investigation into the clinical utility of CMD stratification based on this novel CMD endotype-based classification. Furthermore, one unresolved aspect of this CMD classification is how to interpret patients with preserved CFR but elevated hyperemic MVR, as a unified consensus on whether this physiological profile should be classified as CMD has not yet been established. Although elevated MVR is generally considered to reflect structural abnormalities of the coronary microcirculation, the mechanisms and clinical significance of isolated MVR elevation remain uncertain and may partly relate to methodological factors such as the site of assessment [54, 55]. Recent hemodynamic analyses suggest that patients with preserved CFR but elevated hyperemic MVR may exhibit favorable systemic vascular properties, questioning whether isolated hyperemic MVR elevation truly represents CMD [95]. Whether this endotype reflects a benign adaptive state or carries prognostic relevance remains to be determined.

Building on this conceptual foundation, recent clinical studies have begun to apply this endotype-based classification to assess differential treatment responses according to CMD endotype. The ChaMP-CMD study was a prospective, endotype-blinded, randomized crossover trial that demonstrated the potential benefit of physiology-stratified anti-ischemic therapy using amlodipine and ranolazine in patients with impaired CFR (< 2.5) [38]. As a primary finding, patients with CMD showed greater improvement in exercise time and symptoms in response to treatment compared to those without CMD, suggesting that the presence of CMD may help identify individuals more likely to benefit from such therapy. A secondary analysis of this study revealed that, although both “structural” and “functional” CMD endotypes demonstrated improved exercise tolerance with both agents, the extent of benefit appeared to differ by endotype. Patients with “structural” CMD showed a greater numerical increase in exercise time in response to amlodipine compared to ranolazine (mean difference in delta, 46 s [95% CI, − 2 to 93 s]; *P* = 0.056). In contrast, patients with “functional” CMD exhibited a similar improvement in exercise time with both agents (mean difference in delta, 3 s; *P* = 0.86). This endotype-oriented approach may offer a practical framework for evaluating therapeutic efficacy and personalizing treatment in CMD. Furthermore, integrating novel physiological indices of vasodilator reserve and microvascular resistance, such as MRR and mMR, may extend the applicability of this framework beyond INOCA and enable better characterization of CMD and refined risk stratification across a broader spectrum of cardiovascular diseases.

### Future perspectives — expanding the role of IPA beyond INOCA

CMD extends beyond the context of INOCA and is now increasingly recognized across a broad spectrum of cardiovascular diseases, including hypertrophic cardiomyopathy and cardiac amyloidosis [6]. CMD also frequently coexists with more common conditions such as CAD and heart failure, where it contributes to complex pathophysiology and is associated with adverse clinical outcomes. The potential clinical utility of IPA outside the INOCA population has been demonstrated in several studies. This review highlights the emerging role of IPA in evaluating microvascular function in patients with HFpEF and CAD.

#### The potential utility of evaluating CMD in HFpEF

HFpEF is recognized as a heterogeneous clinical syndrome involving multiple pathophysiological mechanisms, and increasing evidence indicates that CMD plays an important role in its pathophysiology. A cardiac MRI study demonstrated that CFR was significantly lower in HFpEF patients (2.21 ± 0.55) compared with those with hypertensive LVH (3.03 ± 0.71) and healthy controls (3.83 ± 0.73; all *p* < 0.05) [96]. In the PROMIS-HFpEF study, CFR was assessed using Doppler echocardiography, and 75% of HFpEF patients were found to have reduced CFR (< 2.5), which was associated with systemic endothelial dysfunction and higher NT-proBNP levels [97]. Consistently, across studies using different modalities, the prevalence of CMD in HFpEF has been reported to range from 35 to 40% (CFR < 2.0) to as high as 70–80% (CFR < 2.5). This highlights the substantial burden of CMD among HFpEF patients [97]. In recent years, CMD, in conjunction with left ventricular diastolic dysfunction (DD), has been increasingly recognized as an important contributor to the development of HFpEF [6, 98]. CMD shares common risk factors with DD—such as hypertension, diabetes mellitus, and chronic kidney disease—which promote systemic inflammation, oxidative stress, and reduced nitric oxide availability contributing to multi-organ impairment in HFpEF [99–101]. These shared mechanisms may lead to both reduced microvascular density and interstitial fibrosis. Consistent with this, an autopsy study in HFpEF patients demonstrated reduced microvascular density, which was associated with greater interstitial fibrosis [102]. 

In this context, several clinical studies have demonstrated the potential utility of evaluating CMD in HFpEF patients. Taqueti et al. demonstrated a significant inverse relationship between PET-derived CFR and E/e′—an echocardiographic marker of diastolic dysfunction—particularly in patients with CFR < 2.0 [103]. Notably, a detectable troponin was associated with DD only when impaired CFR was also present. This suggests that CMD-related localized myocardial injury may contribute to myocardial interstitial fibrosis and subsequent DD. Furthermore, patients with both impaired CFR (CFR < 2.0) and DD (E/e′ >15) showed a more than 5-fold increased risk of HFpEF hospitalization. These findings support the concept that CMD, when coexisting with or related to DD, may serve as a marker of adverse clinical outcomes.

Taken together, these findings suggest that CMD may, at least in part, contribute to the development and progression of HFpEF by reducing ischemic tolerance and increasing myocardial stiffness, thereby representing an important substrate of the disease. In other words, CMD may constitute an upstream pathophysiological condition preceding the clinical manifestation of HFpEF, and evaluating CMD coexisting with LVDD may be crucial for identifying patients at risk and potentially informing preventive strategies [103]. Regarding the role of IPA in HFpEF, Hong et al. demonstrated that among patients without systolic dysfunction or significant epicardial stenosis, those with both DD and CMD (reduced CFR [< 2.0] and elevated IMR [≥ 25]) experienced higher incidences of cardiovascular death and heart failure hospitalization [104]. While IPA-derived data remain limited, prior evidence suggests that its integration with echocardiographic functional assessment may provide a valuable tool for identifying high-risk patients and improving risk stratification in HFpEF.　However, despite accumulating evidence, the presence and clinical implications of CMD in HFpEF have not yet been adequately addressed in current heart failure guidelines, and its role in guiding therapeutic strategies remains undefined [105–107]. Against this background, to further elucidate the clinical significance of CMD in heart failure, Hibi et al. initiated the DEMAND-HF study (UMIN000048125)—an ongoing, prospective, multicenter registry enrolling patients with heart failure but without functionally significant CAD. This registry aims to clarify the clinical utility and prognostic value of IPA for microvascular function, as well as to elucidate underlying pathophysiological mechanisms through myocardial biopsy and cardiac MRI. Its findings are expected to deepen our understanding of CMD in the context of heart failure with diverse pathophysiological backgrounds.

#### Pathophysiological and diagnostic challenges of CMD in CAD patients

Another important topic is the role of IPA for CMD in the setting of CAD. Although this topic has been recognized for decades, it remains incompletely understood. Several mechanisms may underlie CMD in the setting of obstructive CAD (Type 3 CMD of the clinical classification), including structural alterations such as inward remodeling and capillary rarefaction, which may be driven by low distal perfusion pressure, enhanced endothelin-1–mediated vasoconstriction, and microcirculatory plugging due to plaque debris embolization [17, 108]. Furthermore, these abnormalities may also coexist with CMD of non-epicardial origin, such as Type 1 CMD (primary microvascular disease) or Type 2 CMD (CMD associated with myocardial disease), which can complicate the situation. From a histological perspective, an autopsy study by de Waard et al. demonstrated that the ratio of lumen area to vessel area, as well as vessel density did not significantly differ between vessels with and without obstructive stenosis—either at the capillary or arteriole level [109]. Their findings suggest that the development of microcirculatory impairment appears to occur in parallel, independently of the severity of epicardial stenosis. Meanwhile, Usui et al. reported that the prevalence of optical coherence tomography–detected thin-cap fibroatheroma (TCFA) and plaque rupture, which are markers of plaque vulnerability, increased significantly across IMR tertiles, but not across FFR tertiles [110]. In the highest IMR group (≥ 25 U), TCFA and plaque rupture were observed in 24% and 26% of patients, respectively, compared to 10% and 11% in the intermediate group (15–25 U) and 11% and 8% in the lowest group (≤ 15 U) (*p* < 0.01). Regarding the interaction between CMD and epicardial coronary conditions, the association of coronary spasm with increased hyperemic MVR has been reported [111]. Notably, Suda et al. further demonstrated that patients with epicardial spasm and high IMR experienced poorer clinical outcomes [59]. Collectively, these findings suggest that adverse epicardial conditions and microvascular dysfunction may be linked, potentially through shared cardiovascular risk factors such as inflammation. Although this remains hypothetical, further investigation is warranted to elucidate the interplay between epicardial and microvascular disease in CAD patients.

CMD coexisting with obstructive CAD may synergistically contribute to myocardial ischemia [6, 62, 108]. Furthermore, revascularization alone may be insufficient to fully relieve ischemia if CMD persists, which will be discussed in the next section. One clinical challenge is that FFR can underestimate the severity of CAD under high MVR, as impaired hyperemic flow results in an insufficient pressure gradient across the stenosis [62]. Therefore, a normal FFR value should only be considered reliable if CMD is absent. This diagnostic uncertainty could influence clinical decision-making, although its direct impact on outcomes has yet to be fully elucidated. Conversely, as mentioned before, the presence of obstructive CAD can also complicate the evaluation of CMD, even though IPA is more readily applicable in this population. This overlap of epicardial and microvascular diseases has rendered the clinical significance and therapeutic targeting of CMD in CAD patients a persistent “black box.” To address this, newer indices less dependent on epicardial stenosis have been proposed and may offer broader clinical utility, extending beyond INOCA populations.

#### IPA for CMD in refractory angina after revascularization

Ischemia in the patients with CAD coexisting with CMD may not fully resolve even after revascularization, but addressing the residual CMD would be essential for improving coronary flow and relieving ischemia [112]. “Refractory angina” despite technically successful revascularization remains a major unresolved issue linked to CMD in interventional cardiology [1]. Patients with refractory angina suffer from impaired quality of life and often undergo repeat coronary angiography. Previous studies have reported that 20–30% of patients continue to experience anginal symptoms despite anatomically successful PCI [113, 114]. CMD is likely to contribute to a substantial proportion of this persistent myocardial ischemia. Li et al. reported that patients with recurrent angina after PCI exhibited lower CFR and higher IMR, which are indicative of “structural” CMD, compared to asymptomatic patients [115]. Additionally, long-term data from the COVADIS registry showed that a history of CAD in patients with MVA was an independent predictor of adverse outcomes, primarily due to unstable angina (HR 2.032, 95% CI 1.132–3.147, *p* = 0.001) [116]. These findings suggest that residual ischemia caused by CMD after revascularization remains a challenging clinical issue. Although definitive treatment strategies for CMD are yet to be established, its accurate identification through physiological assessment is increasingly recognized as a key component of optimal patient management in this setting. In line with this, the current international guideline has upgraded the recommendation for invasive coronary functional testing, including IPA for coronary microcirculation, to Class IB in patients with persistent or recurrent angina after PCI [1]. Accordingly, IPA may serve as a valuable tool not only for diagnosis but also for guiding future therapeutic strategies in this unmet clinical need.

#### The prognostic significance of IPA for CMD in patients with CAD

Accumulating evidence from IPA has demonstrated that evaluation of CMD among patients with CAD may provide valuable prognostic information, particularly with regard to atherosclerotic cardiovascular events. For instance, Lee et al. reported that among 230 patients with intermediate stenosis and negative FFR (> 0.80), those with both low CFR (≤ 2.0) and high IMR (≥ 23 U) had the highest incidence of adverse events—including death, MI, and any revascularization, especially in non-target vessels [35]. DEFINE-FLOW, a prospective multicenter registry, enrolled 430 patients with stable intermediate coronary lesions (≥ 50% diameter stenosis) and assessed them using Doppler-based physiology [117]. Patients with normal FFR (> 0.80) but reduced CFR (< 2.0) had higher rates of adverse events compared to those with preserved FFR and CFR. This group also showed numerically higher rates of cerebrovascular events and non-target vessel MI, although the findings were inconclusive due to a low overall event rate and limited two-year follow-up. Like these, several previous studies have suggested that IPA-based CMD assessment may be a useful tool for risk stratification in patients with functionally non-significant epicardial stenosis, where such evaluation is more reliably applicable. Meanwhile, the prognostic significance of IPA for CMD in patients with functionally significant CAD remains unclear, partly due to technical limitations, although some studies using indirect methods of CMD evaluation—such as post-PCI or non-culprit vessel assessments—have suggested the clinical utility of IPA even in those with obstructive CAD [60, 93, 118]. 

In this regard, emerging indices such as MRR and mMR—which evaluate microvascular function independently of epicardial disease—may provide valuable tools for future research. In patients with moderate coronary stenoses, Rasmussen et al. showed that a lower pre-procedural MRR was associated with greater improvement in angina symptoms and hyperemic myocardial perfusion after revascularization, indicating its potential to identify patients who may benefit from the procedure [119]. As mentioned earlier, Boerhout et al. reported that across a wide spectrum of CAD severity, including both non-significant and significant epicardial disease, abnormal MRR (≤ 3.0) was independently associated with an increased risk of MACE over five-year follow-up (HR 1.90; 95% CI 1.17–3.08; *p* < 0.005) and of target vessel failure (TVF) (HR 1.94; 95% CI 1.08–3.49; *p* = 0.017) [79]. Notably, in vessels with functionally significant epicardial disease (FFR < 0.75), MRR—but not CFR—was significantly associated with both MACE and TVF over five years. These results indicate the potential clinical utility of IPA as a means of evaluating microvascular function and highlight the need for future studies to establish its role in guiding therapeutic decision-making in patients with CMD and CAD.

## Conclusion

IPA provides a unique opportunity to evaluate coronary microvascular function, offering mechanistic insight into patient-specific pathophysiology and demonstrating prognostic utility in various cardiovascular diseases. As discussed in this review, IPA-derived indices are still evolving and are expected to play an important role in the diagnosis, risk stratification, and therapeutic guidance of CMD across a wide range of clinical settings.
